# Neurotransmitters and molecular chaperones interactions in cerebral malaria: Is there a missing link?

**DOI:** 10.3389/fmolb.2022.965569

**Published:** 2022-08-24

**Authors:** Michael Oluwatoyin Daniyan, Funmilola Adesodun Fisusi, Olufunso Bayo Adeoye

**Affiliations:** ^1^ Department of Pharmacology, Faculty of Pharmacy, Obafemi Awolowo University, Ile-Ife, Osun State, Nigeria; ^2^ Drug Research and Production Unit, Faculty of Pharmacy, Obafemi Awolowo University, Ile-Ife, Osun State, Nigeria; ^3^ Department of Biochemistry, Benjamin S. Carson (Snr.) College of Medicine, Babcock University, Ilishan-Remo, Ogun State, Nigeria

**Keywords:** neurotransmitters, molecular chaperones, cerebral malaria, *P. falciparum*, cytoadherence, sequestration, inflammation

## Abstract

*Plasmodium falciparum* is responsible for the most severe and deadliest human malaria infection. The most serious complication of this infection is cerebral malaria. Among the proposed hypotheses that seek to explain the manifestation of the neurological syndrome in cerebral malaria is the vascular occlusion/sequestration/mechanic hypothesis, the cytokine storm or inflammatory theory, or a combination of both. Unfortunately, despite the increasing volume of scientific information on cerebral malaria, our understanding of its pathophysiologic mechanism(s) is still very limited. In a bid to maintain its survival and development, *P. falciparum* exports a large number of proteins into the cytosol of the infected host red blood cell. Prominent among these are the *P. falciparum* erythrocytes membrane protein 1 (PfEMP1), *P. falciparum* histidine-rich protein II (PfHRP2), and *P. falciparum* heat shock proteins 70-x (PfHsp70-x). Functional activities and interaction of these proteins with one another and with recruited host resident proteins are critical factors in the pathology of malaria in general and cerebral malaria in particular. Furthermore, several neurological impairments, including cognitive, behavioral, and motor dysfunctions, are known to be associated with cerebral malaria. Also, the available evidence has implicated glutamate and glutamatergic pathways, coupled with a resultant alteration in serotonin, dopamine, norepinephrine, and histamine production. While seeking to improve our understanding of the pathophysiology of cerebral malaria, this article seeks to explore the possible links between host/parasite chaperones, and neurotransmitters, in relation to other molecular players in the pathology of cerebral malaria, to explore such links in antimalarial drug discovery.

## Introduction

Malaria remains one of the disturbing public health challenges and a major vector-borne transmitted infection worldwide (Goldberg and Cowman, 2010; Boel et al., 2012; Khanam, 2017; WHO, 2021). The causative pathogen is *Plasmodium* species, five of which are known to infect human, namely, *Plasmodium falciparum, P. vivax, P. ovale, P. malariae,* and P. *knowlesi*, with *P. vivax* and *P. falciparum* known to cause the most lethal infection (Schiess et al., 2020; WHO, 2021). Its complex life cycle, spanning two hosts, human, and female *Anopheles* mosquito, and unabated transmission, has resulted in the high rate of infections, morbidity, and mortality (Nkumama et al., 2016; Khanam, 2017; WHO, 2021). Malaria is most common in rural, indigenous, and impoverished zones of Africa, Asia, and the tropics of America (Monteiro et al., 2014; Talapko et al., 2019). Prior to the advent of the COVID-19 pandemic, the fight against malaria infection appears to have been gaining success with noticeable decreases in the number of reported infections and deaths when compared to the previous year (Daniyan, 2021). However, the disruption in all malaria intervention areas, including prevention, diagnosis, treatment, elimination, and surveillance, occasioned by the pandemic, has led to marked increases in reported cases and associated mortality (Daniyan, 2021; WHO, 2021). The year 2021 world malaria reports estimated 241 million malaria cases and 627,000 deaths, representing 14 million more cases and 69,000 more deaths in 2020, with about 7.5% of these deaths linked to COVID-19 associated disruption (WHO, 2021). Therefore, the dynamics of malaria disease management has been altered by COVID-19 pandemic, and without a more concerted effort, and new strategies to arrest the upsurge, the gains of yesteryears may soon be completely wiped out.

Cerebral malaria (CM) is a deadly complicating manifestation of severe *P. falciparum* malaria with fast-rising fatal neurological syndromes and a high rate of mortality among children from sub-Saharan Africa (Desruisseaux et al., 2010; Grau and Craig, 2012; Shikani et al., 2012). Cerebral malaria occurs in about 1% of all *P. falciparum* infections and has a high mortality rate of 15%–25% (Shikani et al., 2012; WHO, 2021) leaving its surviving subjects with acute or long-term physical disability and neurological syndrome even post-treatment of the infection (Kihara et al., 2006; Grau and Craig, 2012; Hora et al., 2016). These manifestations differ in children and adults and vary depending on the onset of severe symptoms, including coma and status epilepticus, which could propel focal sequelae, impaired movement, hyperactivity, and inappropriate speech or vision (MacCormick et al., 2014). For instance, CM is not common in adults in sub-Saharan Africa due to acquired immunity during childhood attributable to high malaria transmission rates. In Southeast Asia on the other hand, where the transmission rate is lower and consequently not enough to lead to the development of immunity, CM is more common in adults and older children (Sahu et al., 2015; Sierro and Grau, 2019a). There are differences in CM-related organ dysfunction between adults and children (Sahu et al., 2015; Sierro and Grau, 2019a). While dysfunction in children is almost exclusively neurological, adults experience other organ dysfunctions, such as renal and respiratory dysfunction, in addition to neurological dysfunction (Sahu et al., 2015). Months or years after CM, neuropsychiatric manifestations and disabilities can set in (Monteiro et al., 2014). Meanwhile, despite the undeniable role of increased parasitemia, the levels of parasitemia are not necessarily the determinant for developing the disease, as there is no clear association between particular parasite features and CM (Storm and Craig, 2014a). However, CM is likely to occur in the absence of adequate antimalarials, and to a greater extent among the non-immune people (Bruneel, 2019), necessitating the need for adequate, affordable and accessible health facilities with approved medicine. Unfortunately, the extremely complex aetiology of CM has not been fully elucidated, and thus limiting our understanding of the pathologic mechanisms of CM (Bruneel, 2019; Sierro and Grau, 2019b). Nevertheless, there is a prevailing asymptomatic parasitaemia and certain non—specific pathological features among CM patients presenting with coma (Hora et al., 2016; Idro et al., 2016). In addition, lack of accurate and early diagnosis may have also contributed greatly to limited specificity and knowledge of the infection. Moreover, the available but limited post-mortem histological studies have not vividly explained the processes in the brain, identified the key players, nor shed light on how to ameliorate the aftermath effect of CM, necessitating the need for new and improve approaches to study CM (Sierro and Grau, 2019b). Therefore, despite increasing volume of scientific information on CM, our understanding of the pathophysiologic mechanism(s) of CM is still very limited.

Meanwhile, in a bid to maintain its survival and development, *P. falciparum* exports a large number of proteins into the cytosol of the infected host red blood cell (iRBC) (Acharya et al., 2007; Liu and Houry, 2014). Prominent among these are the molecular chaperones of the heat shock proteins family. Functional activities and interaction of these proteins with one another and with recruited host resident proteins are critical factors in the pathology of malaria infection (Banumathy et al., 2002; Pavithra et al., 2007; Daniyan et al., 2019b). Detailed reviews on the activities of these chaperones and their potential as drug targets have been presented (Shonhai, 2010; Daniyan and Blatch, 2017; Daniyan et al., 2019b; Shonhai et al., 2021). Furthermore, several neurological impairments, including cognitive, behavioral, and motor dysfunctions, are known to be associated with CM (Oluwayemi et al., 2013; Monteiro et al., 2014). Available evidence has implicated glutamate and glutamatergic pathways in CM, coupled with a resultant possible alteration in nitric oxide (NO), serotonin (5-HT), dopamine, norepinephrine, and histamine production (Roy et al., 1993; Enwonwu et al., 1999; Rubach et al., 2015; Yeo et al., 2015; Kempaiah et al., 2016; Oliveira et al., 2017). While seeking to improve our understanding of the pathophysiology of CM, this article seeks to explore the possible links or interactions between host/parasite chaperones, and neurotransmitters, in relation to other molecular players in the pathology of CM, with a view to exploring possible functional relationships in antimalarial drug discovery.

## Pathogenesis of cerebral malaria

There is currently no complete understanding of the pathogenesis of CM. Although some hypotheses, such as that of mechanical obstruction of microvessels (sequestration of parasitized erythrocytes), and that of the release of copious amounts of pro-inflammatory cytokines, have been put forward for the pathogenesis of CM, they do not fully account for disease progression, prognosis, and outcome (van der Heyde et al., 2006; Storm and Craig, 2014a; Dunst et al., 2017; Schiess et al., 2020). The vascular occlusion or sequestration hypothesis is based on the sequestration of iRBCs into the brain capillary endothelia, resulting in microvascular blockage, loss of blood, tissue hypoxia, blood-brain barrier (BBB) compromise, and finally, CM (Storm et al., 2019; Schiess et al., 2020). Sequestration of the parasitized cells is seen as a survival strategy *via* immune evasion used by the parasites to avoid the removal of the cells by the spleen (Amante et al., 2010; Sierro and Grau, 2019a). Moreover, the somewhat hypoxic environment of venous blood is ideal for the growth of the parasites while protecting them from being destroyed by the spleen (Newton et al., 2000). Human CM post-mortem is characterized by swelling of cerebral capillaries and venules containing parasitized and non-parasitized erythrocytes (Newton et al., 2000; Sierro and Grau, 2019a), and platelets (Sierro and Grau, 2019a). Sequestration occurs to different extents in the various vital organs and the severity and prognosis of the disease have been linked to the size of the sequestered biomass (Newton et al., 2000; Ponsford et al., 2012), which cannot be deduced from peripheral parasite counts (Newton et al., 2000). Meanwhile, the binding of sequestered iRBCs to the endothelium is made possible by the *P. falciparum* erythrocytes membrane protein I (PfEMP1), ensuring an increased affinity of iRBCs to various receptors, notably, cytokine-activated endothelial protein C receptor (EPCR) and intercellular adhesion molecule-1 (ICAM-1) on the brain endothelial cells (Storm and Craig, 2014b; Nishanth and Schlüter, 2019; Storm et al., 2019). This is followed by the activation of thrombocytes to stimulate adhesion of iRBCs to one another, forming clots and inducing the non-iRBCs rosettes, hence, restraining blood flow, aggravating microvascular obstruction and tissue hypoxia (Storm and Craig, 2014b; Nishanth and Schlüter, 2019; Schiess et al., 2020), and eventual compromise of the BBB integrity (Ponsford et al., 2012; Rosenberg, 2012; Shimizu et al., 2018).

The inflammatory hypothesis is based on the release of toxic products by *P. falciparum*, causing an imbalance in systemic inflammatory responses, which are exacerbated due to sequestration and cytoadherence of iRBC (Storm and Craig, 2014b; Plewes et al., 2018). The resultant surge in the release of pro-inflammatory cytokines by macrophages, such as tumor necrosis factor-α (TNF-α), interleukin-B1 (IL-B1), and interleukin 10 (IL-10), amplify inflammation and BBB breakage by producing reactive oxygen species (ROS) and nitric oxide (NO) into the circulation (Bruneel, 2019; Schiess et al., 2020), resulting in impaired erythropoiesis and fever (Milner et al., 2014; Milner, 2018; Sierro and Grau, 2019b). On the other hand, these macrophages can as well release interferon-γ which aids the expression of surface proteins such as *Pf*EMP-1, histidine-rich proteins (HRPs), ring erythrocyte surface antigen (RESA) in iRBCs and increase the formulation of adhesion molecules on endothelial cells to aid binding of surface proteins, thereby proliferating vascular permeability in several organs (Sierro and Grau, 2019b). These organs express several adhesion molecules, including cytokine-activated endothelial protein C receptor (EPCR), intercellular adhesion molecule-1 (ICAM-1), thrombospondin, and vascular cell adhesion molecule-1 (V-CAM-1) (Storm and Craig, 2014b; Storm et al., 2019), which further propels the endothelial cell to express several chemokines (Dunst et al., 2017; Nishanth and Schlüter, 2019). Also, *P. falciparum*-induced platelets are capable of binding to iRBCs, endothelial cells, and rosettes to promote immune activation by binding parasite-derived molecules *via* their toll-like receptors to further induce cytokines and chemokines (Bruneel, 2019). A detailed review of the roles of cytokines and chemokines in the pathogenesis of CM has been presented (Dunst et al., 2017). Following the multifunctional involvement of platelets in sequestration, humoral response, and endothelial dysfunction in both hypotheses, further investigation of their roles might pave way for more robust drug intervention.

## The roles of chaperones in cerebral malaria

The life cycle of the *Plasmodium* parasite spans two hosts, mosquito and human. The malaria parasite especially requires a robust adaptation system to cope with the stress of existence in two thermally different hosts, viz: the cold-blooded mosquito vector and warm-blooded humans. This coupled with intermittent febrile events in infected humans predisposes the parasite to experience thermal variations that would be stressful to the organism’s continued survival and growth. To easily adapt to varying physiological changes within these hosts, the malaria parasite expresses a large number of proteins, including molecular chaperones, some of which are exported to aid hosts invasion, facilitate cytoadherence, promote pathogenicity, and establishment of clinical malaria infection (Bozdech et al., 2003; Maier et al., 2008, 2009; Pallavi et al., 2010; Spielmann and Gilberger, 2010; Montagna et al., 2012; Liu and Houry, 2014). Molecular chaperones play critical roles in ensuring that newly synthesized proteins are functional, through proper folding, and where applicable, facilitate their subsequent trafficking to desired destinations and refolded to their native three-dimensional conformations (Hartl, 1996; Smith et al., 1998; Hartl and Hayer-Hartl, 2002; Pallavi et al., 2010). Furthermore, they facilitate the assembly of multi-protein complexes, maintain surveillance on cellular protein quality, and ensure that irreparably damaged proteins are timely handed over for degradation (Hartl, 1996; Hartl and Hayer-Hartl, 2002; Pallavi et al., 2010). They are known to function independently or in association with one another, forming a multi-functional network (Acharya et al., 2007; Pavithra et al., 2007). Generally, molecular chaperones of the heat shock protein (Hsp) superfamily, including Hsp90, Hsp70, Hsp60, Hsp40, and small Hsp, are important in this regard. Among the well-known functional networks involve the functional relationship between Hsp90 and Hsp70 and between these and their counterpart Hsp40 co-chaperones (Chua et al., 2014; Pesce and Blatch, 2014). The functional features of these plasmodial heat shock proteins, functional interplay, and networks of interactions, as well as their potential application in antimalarial drug discoveries, have been presented (Pavithra et al., 2007; Shonhai, 2010; Daniyan and Blatch, 2017; Daniyan et al., 2019b; Shonhai et al., 2021).

Meanwhile, the analysis of potential host-parasite protein-protein interactions has shown that the expression of heat shock proteins is important for efficient PfEMP1 presentation, and thus cytoadherence and sequestration (Rao et al., 2010). The PfEMP1 is a virulence factor encoded by *var* genes and available evidence has shown that the expression of DC8 and DC13 var genes is associated with cerebral malaria (Avril et al., 2013). Its trafficking to the surface of infected erythrocytes involves a network of proteins complex (Wickert et al., 2003; Acharya et al., 2007; Pavithra et al., 2007; Spycher et al., 2008). The PfEMP1 plays a critical role in malaria pathogenesis, cytoadherence, and immune evasion and has been presented as a potential drug target (Pasternak and Dzikowski, 2009; Hviid and Jensen, 2015; Bull and Abdi, 2016). Another virulence factor of importance to the establishment of CM is *P. falciparum* histidine-rich protein II (PfHRP2). The PfHRP2 is known to be transported into the cytosol of infected erythrocytes (Howard et al., 1986), compromised the brain endothelial barrier, promote CM pathogenesis (Pal et al., 2016), caused vascular leakage, and exacerbate CM (Pal et al., 2017). PfHRP2 is also involved in the protection of *P. falciparum* parasite from the toxic effects of heme by aiding neutralization of heme (Huy et al., 2003). Therefore, the combined activities of PfEMP1 and PfHRP2 are critical for the establishment of CM. Also, it has been demonstrated that the ability of human Hsp70 (HSPA1A) to regulate nuclear factor–kappa B (NF-κB) signaling and production of proinflammatory cytokines (such as IL-1β, IL-6, and TNF-α), can be suppressed by intraleukocytic hemozoin (*Pf*Hz). Interestingly, this suppression can be reversed in the presence of glutamine, which upregulates human Hsp70, thereby promoting the activation of NF-κB, and attenuation of overexpression of proinflammatory cytokines (Kempaiah et al., 2016). These findings suggest that suppression of functional activities of human Hsp70 is critical for the establishment of cerebral malaria. However, with their ubiquitous nature and multifunctional activities, as well as the involvements of human heat shock proteins, which are often recruited into the cytosol of iRBC (Banumathy et al., 2002), in nervous system activities (including neurotransmission and neuroprotection), and in modulation of the activities of NF-kB (a critical factor in CM pathology) (Stetler et al., 2010; Fusella et al., 2020), these findings show that more are still yet to be unraveled on the importance of host and plasmodial heat shock proteins in the pathogenesis of cerebral malarial.

## The roles of neurotransmitters in cerebral malaria

Neurotransmitters are critical components of central and peripheral nervous systems. They are signaling molecules that are key players in the abilities of nerve cell, or neuron, to efficiently conveys information both electrically and chemically (Rangel-Gomez and Meeter, 2016). Functionally, neurotransmitters can be classified as excitatory and inhibitory neurotransmitters, neuromodulators, and neurohormones. The excitatory neurotransmitters, such as glutamate, acetylcholine, histamine, dopamine, and norepinephrine, induce or motivate target cells to take action by promoting the generation of the action potential. The inhibitory neurotransmitters function to decrease the chances of target cells initiating actions. Examples are dopamine, serotonin, and gamma-Aminobutyric acid (GABA). The neuromodulators, such as dopamine, acetylcholine, serotonin, norepinephrine, and histamine, can send messages to several neurons simultaneously. Finally, neurohormones, such as oxytocin and vasopressin, induce hormonal activities (Hyman, 2005; Rangel-Gomez and Meeter, 2016; Sheffler et al., 2022). Neurotransmitters can also be classified based on their chemical and molecular properties. They are monoamines (dopamine and norepinephrine); peptides (somatostatin and opioids); purines (adenosine triphosphate); and amino acids (glutamate and glycine). Also, some gaseous substances, such as nitric oxide, and endogenous substances, such as tryptamine and phenethylamines, have been shown to function as neurotransmitters (Rangel-Gomez and Meeter, 2016; Sheffler et al., 2022).

The neurodegenerative abnormalities that are consistent with the progression of cerebral malaria are likely to result from the interplay between specific neurotransmitters at various nerve terminals and their receptors. Available reports have shown that these baseline biochemical changes may lead to deteriorations in the histoarchitectural integrity of localized areas of the brain, especially those concerned with the coordination of cognitive, motor, and neurobehavioural activities (Roy et al., 1993; Cha et al., 1998; Suda et al., 2008; Jamwal and Kumar, 2019). In addition, the interplay between neurotransmitters with specific receptor molecules and associated protein expressions has been implicated in the complex cascade mechanisms of cerebral malaria. For instance, dopamine is known to play a central role in modulating cognitive functions, which may be linked to specific dopamine receptor signaling (Cropley et al., 2006). Specifically, the expressions of dopamine D2 receptor within the striatal medium spiny neurons (MSNs) are involved in the flexibility of human cognitive functions (van Holstein et al., 2011), and its defective signaling may alter metabolic activities within the brain while also taking a deregulatory toll on certain neural functions (Bhatia et al., 2022). Interestingly, the progression of cerebral malaria has been shown to trigger overexpression of dopamine D1 and D2 receptors (Kumar and Babu, 2020). The pathological consequence associated with defective signaling of dopamine receptors is the alteration of the structural and functional histoarchitecture of striatal MSNs (Rangel-Barajas et al., 2015). In addition, while excessive dopamine utilization within the prefrontal cortex was found to be consistent with the decline in cognitive functions among experimental animals (Murphy et al., 1996), the neuronal degenerations in the nigrostriatal dopamine signaling pathway can be very detrimental to motor coordination (Krashia et al., 2019). This evidence suggests that dopamine receptors may play an intermediary role between the induction of CM and associated neurodegenerative changes. Therefore, the defective interaction of dopamine with specific dopamine receptors may likely be having a significant implication on the neuropathogenesis of cognitive decline among CM subjects. Moreover, the interference of estrogen with dopaminergic pathways has been used to establish the memory deficits associated with certain neurodegenerative symptoms (Quinlan et al., 2008; Almey et al., 2015; Tozzi et al., 2015; McEwen and Milner, 2017). However, the relationship between sex hormone levels and the molecular pathway of dopamine-dependent neuromodulation in cerebral malaria is unclear. Consequently, the elucidation of these biochemical mechanisms may explain gender-related susceptibility to cerebral malaria. Also, it is possible that the chronic level of oxidative imbalance, occasioned by the presence of highly reactive metabolic by-products of dopamine, such as 3, 4–dihydroxyphenylacetaldehyde, may be involved in the neuroinflammatory pathologic mechanism of cerebral malaria (Miyazaki and Asanuma, 2009; Juárez Olguín et al., 2015; Monzani et al., 2019; Chen et al., 2020).

Furthermore, glutamate signaling is commonly associated with excitatory neurotransmission (Dauvermann et al., 2017). The interaction of glutamate with several receptors at the presynaptic and postsynaptic terminal is known to play important roles in normal brain functioning (Yamaguchi et al., 2011). Of all the L - glutamate receptors, the dense expressions of the N-methyl–D–aspartate (NMDA) receptor within the hippocampus and the cerebral cortex makes it highly critical for mediating learning and memory, or spatial memory activities within the central nervous system (Kumar, 2015). As ionotropic glutamate receptors, the NMDA receptors also confer synaptic plasticity as well as excitotoxic neuronal dysfunctions (Reiner and Levitz, 2018). Apart from the individual functional roles of glutamate and NMDA—glutamate receptors under physiological conditions, their overexpressions have been implicated in the neuropathogenesis of debilitating degenerative conditions (Singh et al., 2019). As a result, potential agents which can function as a specific antagonist of NMDA receptor signaling are currently being explored as ideal drug candidates for the improvement of cognitive decline and other comorbidities associated with cerebral malaria (de Miranda et al., 2017). Also, the metabolic products of glutamate and their baseline interactions with several important biomolecules have been found to play pivotal roles in the initiation and progression of cerebral malaria, and similar to dopamine, metabolic by-products of glutamate oxidation can also worsen the prognosis of cerebral malaria (Kelly and Stanley, 2001; Walker and van der Donk, 2016; Simões et al., 2018). The overstimulation of glutamate release is known to be excitotoxic (Simões et al., 2018), resulting in epileptic seizures commonly manifested in cerebral malaria (Lewerenz and Maher, 2015). Although the exact pathophysiology is unclear, oxidative stress and upregulated mitochondrial dysfunction have been implicated (Atlante et al., 2001). Also, there is an increasing interest in exploring glutamate receptor antagonist as potential neuroprotective agent (de Miranda et al., 2017).

Apart from dopamine and glutamate, available evidence revealed possible alteration in nitric oxide (NO), norepinephrine, histamine, and serotonin (5-HT) (Roy et al., 1993; Enwonwu et al., 1999; Beghdadi et al., 2009a; Miller et al., 2013; Rubach et al., 2015; Yeo et al., 2015; Kempaiah et al., 2016; Oliveira et al., 2017). For instance, the proinflammatory cytokines induce nitric oxide synthase, guanidine triphosphate (GTP) cyclohydrolase I, and indoleamine-pyrrole-2,3-dioxygenase pathways (Busse and Mülsch, 1990; Sakai et al., 1995; Katusic et al., 1998; Dunst et al., 2017). The induction of GTP cyclohydrolase I lead to the generation of tetrahydrobiopterin, a cofactor for nitric oxide synthase and tryptophan hydroxylase (Sakai et al., 1995; Higgins and Gross, 2010), with subsequent generation of nitric oxide and serotonin respectively. Similarly, the induction of indoleamine-pyrrole-2,3-dioxygenase, a regulatory enzyme in the kynurenine pathway, leads to the generation of quinolinic acid, a potent agonist of N-methyl-D-aspartate (NMDA) glutamate receptor (Guillemin et al., 2005; Lugo-Huitrón et al., 2013). Furthermore, enhanced synthesis of histamine has been linked to cerebral malaria-associated pathogenetic processes, including the disruption of BBB, and sequestration of T lymphocytes to cerebral vascular endothelium in mice (Enwonwu et al., 2000; Beghdadi et al., 2009a, 2009b). Also, an increased level of norepinephrine may lead to the potential aggravation of cerebral vasospasm (Zeiler et al., 2014). Therefore, the understanding of the roles of neurotransmitters, and their functional interplay are important not only to better understand pathogenic processes in CM but also for effective drug intervention and drug discovery.

## Possible links among molecular players in CM pathology

**FIGURE 1:**
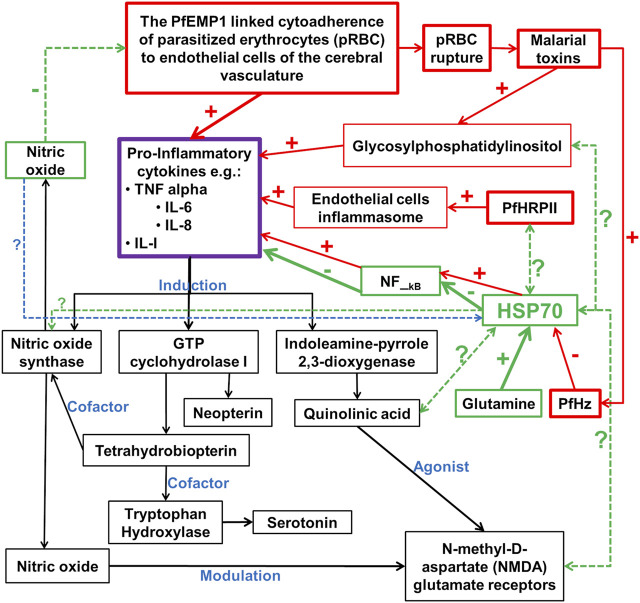
Potential chaperones linked neurotransmitter pathways of the inflammation pathophysiologic mechanism of cerebral malaria. PfEMP1 is *P. falciparum* erythrocyte membrane protein 1; PfHz is hemozoin; PfHRPII is *P. falciparum* histidine-rich protein II; In red boxes are the pro-inflammatory cytokines inducers; In green boxes are the potentially major players in the inhibition of pro-inflammatory cytokines; In black boxes are downstream biochemical cascade potentially linked to the neurological syndrome in cerebral malaria; **-** and **+** signs indicate inhibition and induction respectively; **?** With dotted double or single-faced arrows are potential functional relationships that are yet to be or partially investigated respectively; - and + signs with arrows are reported functional relationships with HSPA1A, but not for plasmodial Hsp70 chaperones.

The ability of iRBC to induce NF-kB-regulated inflammatory pathways in human cerebral endothelium has been demonstrated (Tripathi et al., 2009), indicating that NF-kB signaling has an important role to play in the pathology of CM (Figure 1). The NF-κB transcription factors have been implicated in many key physiological and pathophysiological processes, such as regulation of expression of genes needed for inflammation and immune responses, as well as cell proliferation, and apoptosis. In the central nervous system, the NF-κB is involved in diverse functions, including neuroinflammation. It is constitutively expressed in glutamatergic neurons, such as the cerebral cortex and hippocampus, serves neuroprotective roles, and is implicated in learning and memory. Both canonical and non-canonical NF-kB pathways are involved and serve to regulate gene transcription, including those involved in inflammatory processes, such as cytokines, chemokines, adhesion molecules, and proinflammatory enzymes and transcription factors. NF-κB is also found in glial cells, where its induction leads to the regulation of inflammatory processes that exacerbate neurodegenerating diseases, such as Alzheimer’s disease [reviewed here (Kaltschmidt and Kaltschmidt, 2009; Shih et al., 2015)]. These findings underscore the importance of NF-kB in immune responses to malaria infection (Bąska and Norbury, 2022) and may therefore serve as a critical link between the proposed vascular and inflammation hypotheses of CM pathology (Figure 1). Interestingly, chaperones are known to play functional roles in many processes regulated by NF-kB pathways (Fusella et al., 2020). Such roles depend on the regulated processes as well as on whether these chaperones are constitutively expressed or inducible. For instance, several lines of evidence have validated the inhibitory effects of upregulation of human Hsp70 on the induced expression of TNF-α and IL-6, and the activation of NF-κB (Senf et al., 2008; Wang C.-H. et al., 2017; Lyu et al., 2020) (Figure 1). However, the inhibition of Hsc70 by deoxyspergualin and the siRNA knocked down variant of Hsc70, significantly decreased nuclear translocation and neuronal activity of NF-κB p65. This suggests that Hsc70 may likely be involved in the activation, rather than inhibition of NF-κB (Klenke et al., 2013, 65). In addition, beyond the protein folding and chaperoning, available evidence has also implicated chaperones of the heat shock proteins as critical regulator of normal neuronal physiological functions, including neurotransmission. Essentially, chaperones, especially Hsc70 and human Hsp70 (HSPA1A), and their counterpart DNAJ have been implicated in the vesicular storage of neurotransmitters, release into the synaptic cleft, reforming and recycling of the vesicular membrane, postsynaptic interaction, and G-protein signaling [reviewed here (Stetler et al., 2010)]. For instance, recent evidence has shown that in response to dopamine-induced oxidative assaults on other proteins concerned with neurodegeneration, molecular chaperones are commonly expressed as endogenous mechanisms for curtailing protein aggregation (Webster et al., 2019). These chaperones, including Hsp27 and αβ**—**crystallin (Hayashi et al., 2021), as well as Hsp 40 and Hsp70 (Hu et al., 2021), were able to prevent the aggregation of certain proteins that are associated with the progression of parkinsonism (Hayashi et al., 2021). Interestingly, the induction of Hsp70 molecular chaperone in the dopaminergic neurons has been associated with neurodegeneration (Pastukhov et al., 2013; Smith et al., 2015; Zhang et al., 2018). Nevertheless, the effects of dopamine oxidation on the structural and functional integrities of human and plasmodial Hsp70 and its implication on the pathogenesis of cerebral malaria, remain unclear. However, it is clear that for any CM-mediated neurotransmitter alteration to take place, the malaria parasite needs molecular chaperones for continuous survival and development. Indeed, the presence of Hsp70 in dopaminergic neuron suggest a potential functional relationship. Furthermore, the induction of pro-inflammatory cytokines following cytoadherence and rupturing of pRBC to release malaria toxins (PfHz and glycosylphosphatidylinositol) is linked to the induction of cascade of biochemical pathways that are critical for the establishment of CM (Storm and Craig, 2014b; Storm et al., 2019) (Figure 1). While the induction of nitric oxide synthase and GTP cyclohydrolase I lead to the increased production of serotonin (Katusic et al., 1998), induction of indoleamine-pyrole-2,3-dioxygenase leads to the generation of quinolinic acid, a potent agonist of NMDA receptor (Guillemin et al., 2005; Liu et al., 2015) (Figure 1). Interestingly, nitric oxide is also known to modulate NMDA receptor and may inhibit cytoadherence of pRBC (Hopper et al., 2004). However, the inhibitory effect of PfHz on human Hsp70 can be reversed in the presence of glutamine (Kempaiah et al., 2016) (Figure 1). Therefore, chaperones may be the important link among the many molecular players in the pathophysiology of cerebral malaria (Figure 1). The interrelationship between chaperones and other associated molecular indices (Figure 1) may likely be a promising target for the development of novel drug candidates for the effective treatment of cerebral malaria.

## Potential usefulness of known chaperones targeted small molecule inhibitors in CM

Several reports have shown that heat shock proteins are promising antimalarial drug targets (Pesce et al., 2010; Shonhai, 2010; Shrestha et al., 2016; Daniyan and Blatch, 2017; Daniyan et al., 2019b; Daniyan, 2021). Despite the potential for compensatory upregulation of some heat shock proteins when one is inhibited (e.g., Hsp90 and Hsp70) (Posfai et al., 2018), the essentiality of some members of the heat shock proteins family, their unique functional features, and their ability to form functional networks, not only make their inhibition lethal but that such inhibition could cause a wave of deleterious consequences on the down-stream biochemical processes that these functional networks influence (Pavithra et al., 2007; Daniyan et al., 2019a; Daniyan and Ojo, 2019). Today, small molecule inhibition of plasmodial heat shock proteins has shown promise in reversing *P. falciparum* - induced drug resistance, while also synergizing with traditional antimalarial drugs (Shahinas et al., 2013b; Cockburn et al., 2014; Posfai et al., 2018). Chalcones, polyphenols, terpenoids, alkaloids, and peptides are among the well-tested classes of small molecule inhibitors of heat shock proteins Table 1 [reviewed here (Anokwuru et al., 2021; Daniyan, 2021)]. While there are evidence that some may help in preventing the breakdown of the blood-brain barrier (BBB) (Kam et al., 2012), their potential usefulness in cerebral malaria is largely unexplored.

**TABLE 1:** Features of selected small molecule inhibitors of molecular chaperones with antimalarial/antiplasmodial activities.

Class	Selected member	Biological activities/Mechanisms	Structural formula	References
Chalcones	2,4-Dimethoxy-4′-Butoxychalcone	Acts against *P. falciparum in vitro*, and *P. berghei* and *P. yoelii in vivo*	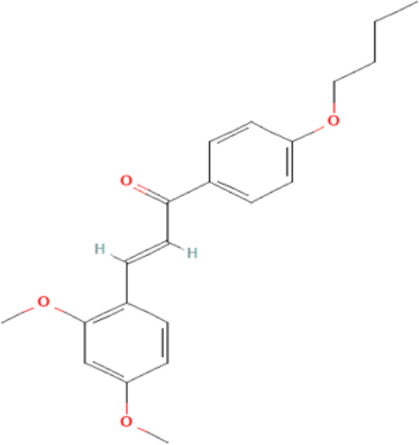	Chen et al. (1997), Salehi et al. (2021)
Polyphenols	Epigallocatechin-3-gallate	Possess antiplasmodial activity, possibly by inhibiting Hsp90 and Hsp70 chaperone and ATPase activities. Neuroprotective	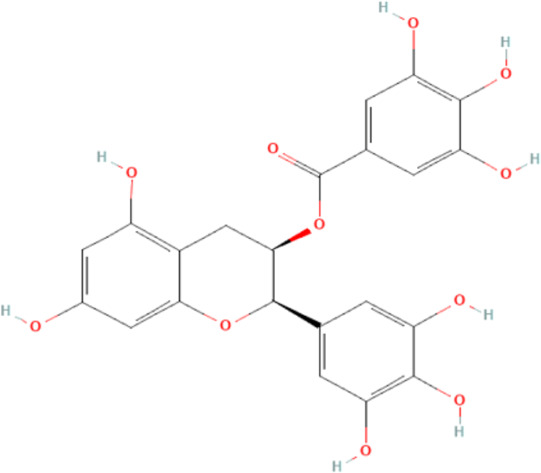	Tran et al. (2010), Zininga et al. (2017b), Youn et al. (2022)
	Curcumin	Modulates NMDA receptors and protects against NMDA and glutamate-induced toxicity. Used as adjuvant in cerebral Malaria	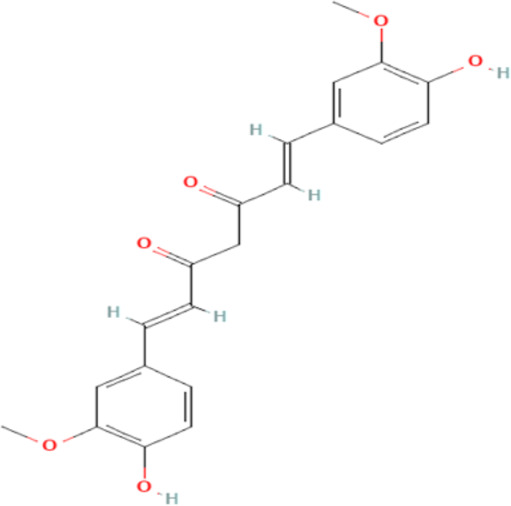	Matteucci et al. (2011), Jain et al. (2013), Wang et al. (2017b), Martí Coma-Cros et al. (2018)
Alkaloids	Quinine	Modulate the expression of some plasmodial proteins, and decrease serotonin production	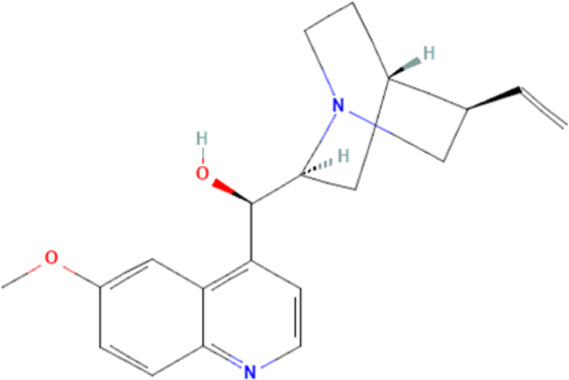	Pussard et al. (2007), Islahudin et al. (2014)
	Geldanamycin	#1C1D1E	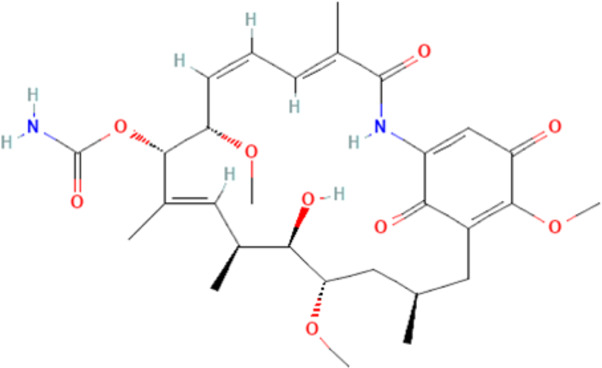	Lu et al. (2002)
		Prevent focal ischemia in the brain, possibly by stimulating heat shock gene transcription		
	^a^17-AAG	Hsp90 inhibitory activities	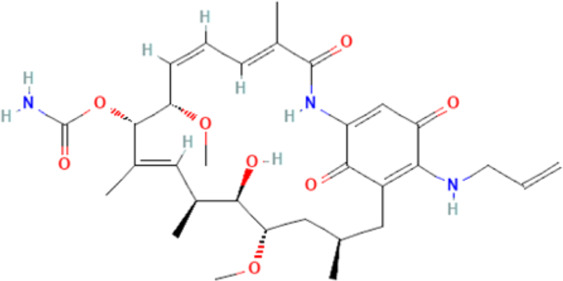	Wu et al. (2010), Shahinas et al. (2013a), Posfai et al. (2018)
	Malonganenone A	Selectively inhibit plasmodial Hsp70	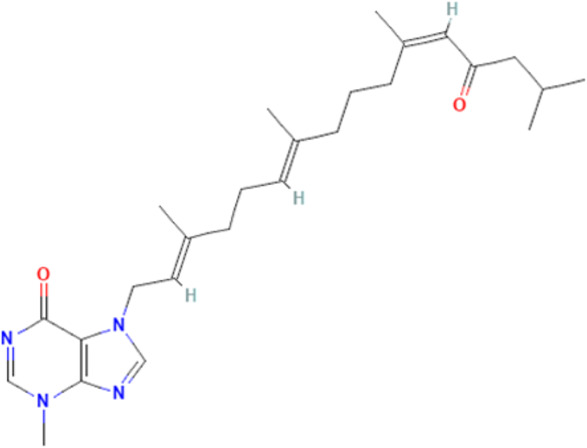	Cockburn et al. (2011), (2014)
Terpenoids	Gedunin	Inhibit Hsp90 and/or induced degradation of Hsp90-dependent client proteins	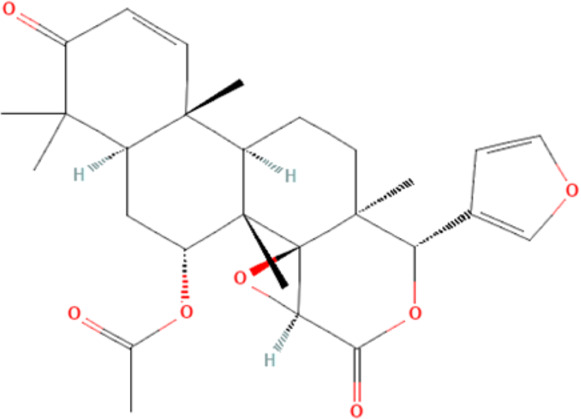	Mackinnon et al. (1997), Brandt et al. (2008), Patwardhan et al. (2013)
	Celastrol	Protects motor neurons from excitotoxicity, possibly by induction of increased Hsp70 expression	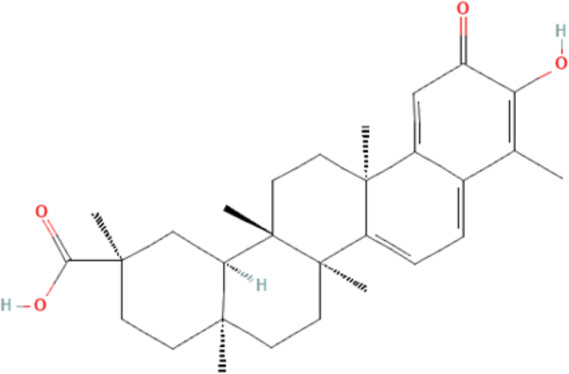	Westerheide et al. (2004), Petrović et al. (2019)
Peptide antibiotic	Polymyxin B	Immunosuppressant, and inhibition of activities of PfHsp70-1	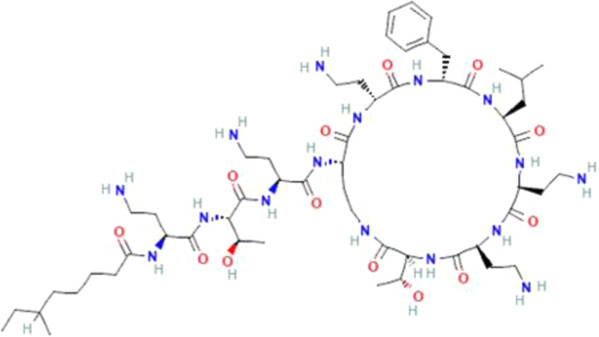	Zininga et al. (2017a)
	Deoxyspergualin	Immuno-suppressant. Modulate Hsc70 activity, primarily targeting trafficking of apicoplast protein in *P. falciparum*	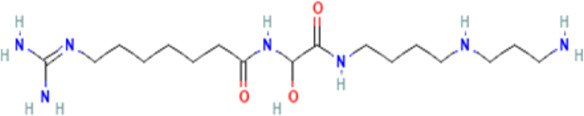	Midorikawa and Haque (1997), Brodsky (1999), Ramya et al. (2007)

a 17-AAG, is 17-allyamino-17-demethoxygeldanamycin.

Chalcones possess several biological activities and have shown promise in antimalarial drug discovery (Singh et al., 2014; Murwih Alidmat et al., 2021; Salehi et al., 2021). The reported potent antimalarial activity of a novel oxygenated chalcone, 2,4-Dimethoxy-4′-Butoxychalcone, against *Plasmodium falciparum in vitro*, and *Plasmodium berghei* and *Plasmodium yoelii in vivo* (Chen et al., 1997), suggest a possible role for chalcone in the management of cerebral malarial. However, an important limitation is their low bioavailability (Mathew et al., 2016; Sinha et al., 2021), necessitating the need for improvement in the design of chalcone derivatives. Polyphenols, on the other hand, can suppress neuroinflammation, as well as promote memory, learning, and cognitive function (Vauzour, 2012; Figueira et al., 2019). They are neuroprotective, possibly *via* neuronal protection against oxidative stress and inflammatory injury (Filosa et al., 2018). While some polyphenols, such as epigallocatechin, daidzein, genistein, equol, and nobiletin, are reported to show high BBB permeability, others like apigenin, and kaempferol, as well as epicatechin, and curcumin showed medium to no permeability (Figueira et al., 2017, 2019; Shimazu et al., 2021). Interestingly, epigallocatechin-3-gallate, an essential polyphenol in green tea, has demonstrated antiplasmodial activity and inhibited both the chaperone and ATPase activity of Hsp90 and plasmodial Hsp70 (Tran et al., 2010; Moses et al., 2015; Zininga et al., 2017b). These reported dual inhibitory activities on Hsp90 and Hsp70 chaperones, coupled with the ability to cross the BBB, and protect neurons (Youn et al., 2022), suggest that epigallocatechin-3-gallate may be a promising candidate in the management of cerebral malaria. In addition, curcumin, another neuroprotective polyphenol (Chin et al., 2013; Wang X.-S. et al., 2017; Subedi and Gaire, 2021), has severally been shown to have potential as adjuvant therapy in ameliorating neurological syndrome in cerebral malarial despite its poor bioavailability (Jain et al., 2013). However, recent evidence has shown that the use of nano-formulated and liposome-incorporated curcumin has the potential to improve the bioavailability and biological activity of curcumin (Dende et al., 2017; Martí Coma-Cros et al., 2018). Moreover, curcumin modulates NMDA receptors and protects against NMDA and glutamate-induced toxicity (Matteucci et al., 2011; Mallozzi et al., 2018; Simões et al., 2018). Therefore, polyphenols are a promising class of small molecules in the management of cerebral malarial.

Meanwhile, alkaloids remain one of the oldest and most popular classes of compounds whose members have found usefulness for decades as antimalarial agents. The most popular member of this group is quinine (Kaur et al., 2009). Quinine, given within the recommended doses, is safe, and at higher doses with proper monitoring, the benefits often outweigh the exaggerated toxicity (White et al., 1982; Pussard et al., 2003). Moreso, quinine, though an old friend, still has a comparable level of effectiveness with newer artesunate despite the reported superiority of parenteral artesunate (Dondorp et al., 2010; Eltahir et al., 2010; Sinclair et al., 2012; Abdallah et al., 2014). However, though available evidence suggests that quinine does not freely cross the BBB (Silamut et al., 1985), quinine uptake into the brain can be increased by inhibition of P-glycoprotein (Pussard et al., 2007). Thus, inhibition of P-glycoprotein may improve the effectiveness of quinine in cerebral malaria through enhanced uptake into the brain. Also, quinine could modulate the expression of some plasmodial proteins, including enolase (PF3D7_1015900), endoplasmic reticulum-resident calcium-binding protein (PF3D7_1108600), chaperonin (PF3D7_1213500), the host cell invasion protein (PF3D7_1027300) and proteins related to redox processes (PF3D7_0827900) (Segura et al., 2014). In addition, quinine has been shown to decrease serotonin production *via* competitive inhibition of tryptophan hydroxylase in the presence of tryptophan (Islahudin et al., 2014), suggesting a potential mechanism of quinine action in cerebral malaria. Other members of the alkaloid class of compounds that have shown antimalarial properties and demonstrated Hsp90 inhibitory activities include ganetespib, harmine, PU-H71, geldanamycin (GA), and its analogs, 17-dimethylaminoethylamino-17-demethoxygeldanamycin (17-DMAG) and 17-allyamino-17-demethoxygeldanamycin (Shahinas et al., 2013a, 2013b; Posfai et al., 2018). The available report shows that geldanamycin could prevent focal ischemia in the brain, possibly by stimulating heat shock gene transcription (Lu et al., 2002). The synergistic ability of PU-H71 with chloroquine (Shahinas et al., 2013b), suggests that these compounds can be combined with existing antimalarial agents to improve treatment outcomes and reduce resistance. Also, a marine prenylated alkaloid, malonganenone A was shown to possess the antimalarial activity and selectively inhibit plasmodial Hsp70 (Cockburn et al., 2014).

Furthermore, terpenoids, which are the most abundant and structurally diverse secondary metabolites, possess a wide range of pharmacological activities, including antimalarial effects and heat shock protein inhibitory activities (Wang et al., 2005; Gabriel et al., 2018; Anokwuru et al., 2021). For instance, gedunin, kotschyins D, celastrol, and fusicoccane have been shown to inhibit Plasmodial Hsp90 and/or induced degradation of Hsp90-dependent client proteins (Westerheide et al., 2004; Brandt et al., 2008; Zhang et al., 2008; Piaz et al., 2012; Patwardhan et al., 2013; D’Ambola et al., 2019). Interestingly, celastrol also induces increased expression of human Hsp70 and protects motor neurons from excitotoxicity (Westerheide et al., 2004; Petrović et al., 2019). It is tempting to postulate that celastrol may find usefulness in cerebral malarial as evidence has shown that increased expression of Hsp70 prevents the production of inflammatory mediators *via* inhibition of NF__kB_, (Kempaiah et al., 2016). In addition, a peptide antibiotic, polymyxin B, and an immunosuppressant, deoxyspergualin, have also demonstrated antimalarial activities (Anokwuru et al., 2021). Polymyxin B showed inhibitory activities on PfHsp70-1 (PF3D7_0818900) and PfHsp70-z (PF3D7_0708800) (Zininga et al., 2017a).

Therefore, while there are limitations, such as limited or inability to cross the blood-brain barrier, and limited to lack of information on the effects of these small molecules on brain neurotransmitters, there is more than enough evidence to propel further research into their potential usefulness in the treatment or as adjuvant therapy to mitigate the deleterious effects of the associated neurological syndrome in cerebral malaria.

## Searching for new drug candidates from natural products

Plants and other natural sources have been the bedrock of drug discovery over the years (Balunas and Kinghorn, 2005; Ahmad et al., 2006; Gurib-Fakim, 2006; Dzoyem et al., 2013). They are not just serving as sources of drug candidates, they have and still are been used in traditional medicine as worthy alternatives to orthodox drugs (Ahmad et al., 2006; Amoateng et al., 2018). Many of the tested small molecules are from these natural sources [reviewed here (Anokwuru et al., 2021; Daniyan, 2021)]. Many medicinal plant products have shown antimalarial, and/or neuroprotective abilities (Kaur et al., 2009; Mahomoodally, 2013; Amoateng et al., 2018; Erhirhie et al., 2021), and may therefore find usefulness in the management of cerebral malarial. Apart from those already mentioned and discussed, Table 2 provided a list of some medicinal plants’ derived compounds with neuroprotective and/or antimalarial activities which may find usefulness in cerebral malarial.

**TABLE 2:** Some medicinal plants’ derived compounds with neuroprotective and/or antimalarial properties.

Compounds	Related biological effects	References
Ginsenoside and ginseng	Antimalarial, anti-inflammatory, antioxidant, antiparasitic	Han et al. (2011), Huang et al. (2019)
Bacopasaponin	Antioxidative, increased cerebral blood flow, neuromodulatory	Aguiar and Borowski (2013), Velaga et al. (2014)
Betulinic Acid	Curtails neuroinflammation by modulating NF-kβ and inhibition of Interleukin -6; Antimalarial	Vijayan et al. (2010)
6-Gingerol	Reduced IL-6, TNF-α, nitric oxide, cerebral cortex lesion; Reversed memory deficit and cognitive impairment	Farombi et al. (2018), Kim et al. (2018), Huang et al. (2019)
Zingerone	Prevents lipid peroxidation and neuroinflammation; Antimalarial	Alam (2018), Ounjaijean and Somsak (2020)
Chrysin	Reduced neuroinflammation; Antimalarial	Nabavi et al. (2015)
L-3-*n*-butylphthalide	Increases expression of glutamic acid decarboxylase, anticonvulsant, antioxidative, and anti-inflammation. Attenuate cognitive decline	Bhatt et al. (2017), Liao et al. (2018), Ye et al. (2018)
Quercetin	Antioxidant, improved motor activity, anti-inflammation; Antiplasmodial	Ganesh et al. (2012), Ezenyi et al. (2014), Singh et al. (2017)
Rutin	Antioxidative, anti-inflammatory, antiapoptotic; Antimalarial	Budzynska et al. (2019), Bhatt et al. (2022)
Baicalein	Modulation of NMDA receptor, ameliorates neuroinflammation, anti-apoptotic, antiexcitotoxic, antipyretic	Hung et al. (2016), Sowndhararajan et al. (2018)
Caffeic acid	Antioxidative, anti-inflammation, decreases neuronal apoptosis, boosts memory; Antimalarial	Fu et al. (2017), Alson et al. (2018), Morroni et al. (2018)
Magnolol	Free radical scavenging, prevents brain infarction and cognitive deficits, anti-inflammation, reduces neuronal apoptosis	Chen et al. (2011), Santos et al. (2021)
kavalactone	Curtails oxidative stress and neuroinflammation, anticonvulsant, anti-anxiolytic, sedative, modulation of GABA receptors	Tzeng and Lee (2015), Alves et al. (2021)
kolaviron	Anti-inflammatory, antioxidative, improved motor and cognitive decline, antiapoptotic, antimalarial	Oluwatosin et al. (2014), Farombi et al. (2019)
Riboflavin	Mitigates oxidative stress, neuroinflammation, and glutamate neurotoxicity; Inhibited hemozoin production, decreases food vacuole size in *P. falciparum*	Akompong et al. (2000), Tripathi et al. (2014), Huang et al. (2022)
Huperzine A	NMDA receptor antagonist inhibits acetylcholine esterase and improves cognition	Zhang (2012), Gul et al. (2019)
Berberine	Prevents learning and memory impairment, heavy metal chelation	Hussien et al. (2018), Wang et al. (2018)
Andrographolide	Inhibit NF-κB activation. Prevent inflammation. Antimalarial	Mishra et al. (2011), Raghavan et al. (2012), Chang et al. (2014)

## Conclusion

Cerebral malaria is a deadly complication of severe *P. falciparum* malaria, with many survivors left to cope with long-term neurological deficits. A thorough understanding of the functional interplay among major molecular players, including molecular chaperones, neurotransmitters, and NF-kB signaling, in the pathology of CM, is critical for the development of a new treatment approach, and a new focus on antimalarial drug discovery. With their ubiquitous nature, multi-functional activities in the central nervous systems, and NF-kB signaling pathways, host heat shock protein chaperones, maybe the critical link amidst these molecular players. However, while functional interactions between host and parasite proteins are well established, the functional interplay between plasmodial heat shock proteins, especially the exported chaperones, and other molecular players (neurotransmitters, NF-kB, etc), requires further investigations.
